# Exploring the connection between parental bonding and smartphone addiction in Chinese medical students

**DOI:** 10.1186/s12888-022-04355-7

**Published:** 2022-11-16

**Authors:** Chunyu Xin, Ning Ding, Nan Jiang, Honghe Li, Deliang Wen

**Affiliations:** Institute for International Health Professions Education and Research, China Medical University, No.77 Puhe Road, Shenyang, Liaoning Province 110122 China

**Keywords:** Smartphone addiction, Parental bonding, Maternal parenting, Paternal parenting, Medical students, Chinese

## Abstract

**Background:**

Smartphone addiction bodes adverse consequences, affecting different populations, including medical students. Parental bonding in childhood had been associated with addiction and recovery in later life. Therefore, this study aimed to examine the associations between parental bonding and smartphone addiction among Chinese medical students.

**Method:**

Binary logistic regressions were used to investigate the associations between parental bonding with mothers and fathers, respectively, and smartphone addiction. Interaction terms of care and protection were included in the models.

**Results:**

A total of 517 medical students were included in the study. The prevalence of smartphone addiction was 48.16% (*n* = 249). The estimated effects of maternal and paternal parenting on smartphone addiction differed. Maternal protection was positively associated with smartphone addiction (OR, 1.046;95% CI, 1.005-1.087), and maternal care enhanced the estimated effect of protection on smartphone addiction. Paternal care was negatively associated with smartphone addiction (OR, 0.954;95% CI, 0.919-0.989).

**Conclusions:**

Chinese medical students with overprotective mothers or with indifferent fathers tended to exhibit traits of smartphone addiction. Further studies on factors influencing the associations between parental bonding and smartphone addiction may pave the way for potential family-oriented interventions for smartphone addiction.

## Introduction

With the rapid development of electronics and information technology, worldwide smartphone mobile subscriptions surpassed 6.5 billion in 2022 and are expected to reach 7.7 billion in 2027 [1]. The multiple features of smartphones [2], such as communications, multimedia, photography, navigation, and internet access, have made our social, cultural, and economic relations much more efficient [3]. However, with the dramatic increase in smartphone users, problems related to smartphone use are also becoming more and more serious [4]. Long duration of [5] and frequent daily [6] smartphone use has led to unprecedented rates of smartphone addiction, which has been characterized by previous studies as the overuse of smartphones that is interfering with the user’s daily life [2]. In fact, prevalence of smartphone addiction has been reported in several countries [7–10], of which one Saudi study reported a proportion reaching up to 66.4% in Arab medical students.

For healthcare professionals, smartphones have become a convenient and portable communication tool and resource, allowing easy access to inter-provider communications, patient resources, medical calculators, and medical information databases [11]. However, this has both its benefits and drawbacks, especially when considering the rise in smartphone addiction [3]. Some evidence of digital addiction and attitudes toward smartphone use in medical students have been presented in past studies [12, 13], with medical students reporting using their smartphones for both personal and educational purposes in the classroom. When smartphone use becomes an addiction that interferes with daily life and with professional activities, a multitude of consequences may occur. Previous studies found relationships between smartphone addiction and physical problems [14], mental disorders [15], as well as social maladjustment [4]. Furthermore, smartphone addiction was found to be associated with poor academic performance among university students [16], and this might further lead to impaired clinical performance [17]. Problematic smartphone use may even compromise medical professionalism [18], causing instability within the healthcare system [19] and potentially compromising patient care. Despite there being data on the negative consequences of smartphone addiction, mechanisms and influences of smartphone addiction still warrant further attention, especially in professional spheres where smartphones are heavily used for communication and educational purposes.

Several studies have been conducted to investigate predictors for smartphone addiction by considering individual factors (gender [20], age [21], personality [22]), family factors (family functioning [23], parental bonding [24]) and social factors (peer relationship [23], social engagement [25]). Of these, family factors have been widely studied in addiction and recovery [26, 27]. Since the family is considered to be the basic unit of any society or culture, it contributes greatly to all aspects of an individual’s development [23]. For example, Korean delinquent adolescents have been reported to have come from troubled families, including those with dysfunctional marital dynamics, family violence, and poor family functioning [28].

Among family factors predicting smartphone addiction, parental bonding had attracted considerable research interest [7]. Parental bonding is defined as a process of performing a social role or behaving to facilitate the offspring’s growth and development [29]. According to attachment theory, developed by Bowlby [30–32] and Ainsworth et al. [33], experiences of parental bonding in childhood which adequately meet children’s needs can help them develop secure representations of self and others. These representations act as “prototypes” or “working models”, constantly influencing children’s expectations, strategies, and behaviors through the whole life. Drawing from attachment theory, Parker et al. developed the Parental Bonding Instrument (PBI), which includes two dimensions: care and protection [34].

The care dimension evaluates the perception of warmth, emotional closeness, and empathy, while the protection dimension evaluates the perception of control, intrusion, and level of independence and autonomy from parents. A longitudinal study from New Zealand found that low parental care and high parental protection during childhood were associated with impaired psychosocial functioning in adulthood, including depression, anxiety, and crime [35]. Subsequently, a study conducted among Chinese college students showed that negative parenting styles, which consisted of parental rejection and overprotection, exacerbated the degree of smartphone addiction [24]. Another study had suggested that addiction can be a coping mechanism for lack of relational intimacy and demonstrated that parental care could positively influence children’s self-identification and help them to forge intimate relationships with others [36]. The self-medication hypothesis suggests that children, especially those who have experienced traumatic upbringings involving parental neglect [37], could eventually become addicted to smartphones to cope with existing strains of family dysfunction [37, 38]. On the other hand, studies have also revealed that overprotective parental behaviors may increase addictive behaviors in children [39]. This may be partially attributed to the effect of parental overprotection on the development of the child’s autonomy [39], which had been listed as a protective factor for addiction behavior [40]. The conflict between children’s desire for autonomy and parental overprotection may eventually lead to addictive behaviors such as problematic smartphone usage [39].

Despite the aforementioned research on care and protection, studies have indicated that these two aspects should not be analyzed separately, because they interact with each other when affecting children’s addiction. For example, a previous study revealed that the combination of parental overprotection and low parental care (known as the affectionless control parenting style) was associated with perpetrating cyberbullying [41]. A cross-study conducted in Brazilian medical students found that dysfunctional parenting styles, including affectionless control (low care and high protection), affectionate constraint (high care and high protection), and neglectful parenting (low care and low protection) were associated with mental anxiety, depression, suicidal ideation, and low self-efficacy [42]. Another implication from the literature is that the effects of maternal and paternal parenting on children’s psychosocial functioning may differ [42]. For instance, only maternal and not paternal overprotection was found to be associated with mood disorders in a study of European countries [43]. In China, mothers and fathers participate in child rearing and in their children’s education to differing degrees [44]. Nevertheless, few studies have separately examined the associations between parental bonding and smartphone addiction for mothers and for fathers.

In response to the aforementioned significance of parental bonding in childhood and the negative effects of smartphone addiction on medical students, the objective of this study was to examine the relationship between parental bonding—separately for mothers and for fathers—and smartphone addiction among Chinese medical students.

## Materials and methods

### Participants

This cross-sectional study was carried out at China Medical University in Shenyang, Liaoning province, during November 2019. Since the dependent variable, namely smartphone addiction, was a binary variable in our study, we used the following formula to estimate the sample size [45]: $n={\left(\frac{{\textrm{Z}}_{\upalpha}}{\updelta}\right)}^2\times \textrm{p}\times \left(1-\textrm{p}\right)$, where n is the sample size; α is the level of significance, 0.05 in our study, and so Z_α_, the critical value of z-distribution, was 1.96; δ, the marginal error, was 0.05 in this study; p, the population proportion of smartphone addiction, was set to be 50% according to the literatures [46, 47]. The calculation showed that the required sample size was 385. Accounting for potential invalid data and low response rates during the final examination season, we surveyed the entire third-year medical student population (*n* = 969) from China Medical University.

Inclusion criteria included: all full-time third-year clinical medical students at China Medical University; and enrolment was in the year 2017. Exclusion criteria included: part-time students or students enrolled for partial credit; transfer students from a program other than clinical medicine; students who have failed a year; and students who have taken time off during medical school for various reasons. A total of 535 students completed the survey, and we excluded 18 invalid responses from the analysis, resulting in 517 valid responses. The online questionnaires were distributed through the platform “Sojump”. Participation was voluntary, and all participants signed an informed consent prior to completing the questionnaire. The study protocol for data collection was approved by the China Medical University Ethics Committee. All methods were carried out in accordance with relevant guidelines and regulations.

## Measures

### Demographics

Information including age, gender, duration of medical program, household registration area (urban/rural), parents’ education levels, parents’ occupations, and annual household income were collected through a demographic questionnaire. Duration of medical program was categorized as either a 5-year bachelor’s degree program or an 8-year bachelor’s and master’s combined program [48]. Household registration area referred to the assigned residence registration system in China and was classified as either “urban” or “rural” [49]. Annual household income was classified into four categories: less than 20,000 Chinese Yuan (CNY), 20,001 to 50,000 CNY, 50,001 to 100,000 CNY, and more than 100,000 CNY.

### Smartphone addiction

The short version of the Smartphone Addiction Scale (SAS-SV) was used to assess students’ smartphone addiction. The SAS-SV, developed by Kwon M et al., is a self-reported scale used to assess smartphone addiction [50] and had been translated into several languages, with validity evidence [2, 8, 51]. The Chinese version of the SAS-SV had demonstrated good psychometric properties [52]. The total score of this 10-item scale ranged from 10 to 60, with a higher score indicating a higher degree of smartphone addiction. According to the scale developers [50], cut-off scores of ≥31 for males and ≥ 33 for females indicated smartphone addiction.

### Parental bonding

The Parental Bonding Instrument (PBI) was used to assess students’ maternal and paternal parenting in this study. The PBI, developed by Parker et al. [34], is a retrospective scale completed separately for mothers and for fathers that measured each parent’s “care” and “protection”. The Chinese version of the Parental Bonding Instrument used in this study includes 23 items, with 12 items in the care dimension and 13 items in the protection dimension, and had previously shown validity evidence in Chinese university students [53]. Participants recalled and evaluated their parental bonding experience in their first 16 years of life on a 4-point Likert scale, with scores of each item ranging from 0 (“very unlike”) to 3 (“very like”). Total scores ranged from 0 to 33 points in the care dimension and 0 to 36 points in the protection dimension. Cronbach’s α was calculated for both maternal and paternal scales in this study.

### Statistical analysis

In the descriptive analyses, means (standard deviation, SD) and frequencies were used to describe continuous and categorical variables, respectively. We used t-tests and χ^2^ tests to compare the differences of the demographics between non-addictive and addictive groups. Logistic regressions were conducted to examine the relationship between parental bonding and smartphone addiction for mothers and fathers, respectively. Subsequently, interaction terms were included to examine interactive effects between care and protection dimensions. Parental bonding variables, individual characteristics (age, gender, duration of medical program, household registration area), and family characteristics (annual household income, mother/father’s education level, mother/father’s occupation) were gradually entered into the logistic regression models to determine the possible effects of individual and family characteristics.

Odds ratios (OR) and 95% confidence intervals (95% CI) were calculated to quantify the relative differences between groups. Marginal effects (ME), specifically average marginal effects (AME), of parental bonding variables were evaluated to quantify incremental differences [54]. AME was derived by calculating the ME for each observed value and averaging over the single ME. In addition, log likelihood, pseudo R^2^, Akaike information criterion (AIC), and Bayesian information criterion (BIC) were reported for each logistic model to assess the fitness of the models. The BIC aims to choose the true model with the growth of sample size, while AIC aims to select the model that minimizes mean squared error of estimation [55]. Smaller AIC or BIC indicates better models. Associations were considered to be significant if a two-tailed *p*-value was < 0.05.

Missing data for household registration, mother’s occupation, father’s occupation, mother’s education level, and father’s education level were replaced using multiple imputations (MI). Although 3-5 sets is usually deemed to be adequate [56], ten imputed data sets were generated in this study. All statistical analyses were conducted using Stata (version 15.0; StataCorp, College Station, TX).

## Results

A total of 535 students completed the survey, and, based on the inclusion and exclusion criteria, we excluded 18 invalid samples from the analysis. In the end, we collected 517 valid study questionnaires, resulting in an effective response rate of 53.35%. The mean age was 20.25 years, and the proportion of males was 37.52% (*n* = 194). Of the total number of participants, nearly half (48.16%, *n* = 249) were considered to have smartphone addiction. The mean scores of paternal care and protection were 23.03 and 9.50, respectively, while mean maternal care and protection scores reached 24.49 and 10.74, respectively. The majority (70.60%, *n* = 365) of students belonged to the 5-year bachelor degree program, and 63.63% (*n* = 329) of the students had urban household registrations. Annual household incomes were mostly 50,000 to 100,000 CNY (41.78%, *n* = 216). Parental educational levels were evenly distributed, and parents were listed as having varying occupations. The Chinese version of the Parental Bonding Instrument showed good reliability in this study. Cronbach’s α was 0.93 for both maternal and paternal scales.

Results from t-tests showed that maternal and paternal care scores for the non-addictive group were significantly higher than those for the addictive group (*t* = 3.77, *p* < 0.001; *t* = 4.60, *p* < 0.001), and maternal and paternal protection scores for the non-addictive group were significantly lower than those for the addictive group (*t* = − 4.17, *p* < 0.001; *t* = − 3.78, *p* < 0.001). There were no significant differences for individual or family characteristics between non-addictive and addictive groups. Table 1 shows detailed characteristics of the study participants.

**Table 1:** Characteristics of participants enrolled in the study

Variables	Total, Mean ± SD or n (%)	Non-addictive, Mean ± SD or n (%)	Addictive, Mean ± SD or n (%)	t or χ^**2**^ value	***p*** value
**Smartphone addiction**					
Smartphone addiction (binary variable)					
No	268 (51.84)	–	–	–	–
Yes	249 (48.16)	–	–	–	–
Smartphone addiction (SAS-SV score)	31.13 ± 10.26	–	–	–	–
**Parental bonding**					
Maternal parenting (PBI-M score)					
Care	24.49 ± 6.20	25.47 ± 6.17	23.44 ± 6.06	3.77	< 0.001
Protection	10.74 ± 6.27	9.65 ± 6.48	11.92 ± 5.82	−4.17	< 0.001
Paternal parenting (PBI-F score)					
Care	23.03 ± 6.55	24.29 ± 6.73	21.69 ± 6.08	4.60	< 0.001
Protection	9.50 ± 6.07	8.53 ± 6.42	10.53 ± 5.49	−3.78	< 0.001
**Individual characteristics**					
Age	20.25 ± 0.77	20.26 ± 0.82	20.23 ± 0.70	0.42	0.674
Gender					
Male	194 (37.52)	97 (36.19)	97 (38.96)	0.42	0.517
Female	323 (62.48)	171 (63.81)	152 (61.04)		
Duration of medical program					
5-year	365 (70.60)	193 (72.01)	172 (69.08)	0.54	0.464
8-year	152 (29.40)	75 (27.99)	77 (30.92)		
Household registration area					
Rural	159 (30.75)	79 (29.48)	80 (32.13)	0.50	0.780
Urban	329 (63.64)	173 (64.55)	156 (62.65)		
Missing	29 (5.61)	16 (5.97)	13 (5.22)		
**Family characteristics**					
Annual household income (CNY)					
≤ 20,000	97 (18.76)	43 (16.04)	54 (21.69)	4.98	0.289
20,000-50,000	119 (23.02)	57 (21.27)	62 (24.90)		
50,000-100,000	216 (41.78)	122 (45.52)	94 (37.75)		
≥ 100,000	41 (7.93)	22 (8.21)	19 (7.63)		
Missing	44 (8.51)	24 (8.96)	20 (8.03)		
Father’s educational level					
Junior high school or below	165 (31.91)	77 (28.73)	88 (35.34)	2.95	0.400
High school	154 (29.79)	86 (32.09)	68 (27.31)		
University or above	158 (30.56)	83 (30.97)	75 (30.12)		
Missing	40 (7.74)	22 (8.21)	18 (7.23)		
Mother’s educational level					
Junior high school or below	177 (34.24)	85 (31.72)	92 (36.95)	2.41	0.493
High school	138 (26.69)	71 (26.49)	67 (26.91)		
University or above	151 (29.21)	82 (30.60)	69 (27.71)		
Missing	51 (9.86)	30 (11.19)	21 (8.43)		
Father’s occupation					
Legislators, senior officials and managers	92 (17.79)	47 (17.54)	45 (18.07)	5.29	0.624
Professionals, technicians and associate professionals	62 (11.99)	38 (14.18)	24 (9.64)		
Clerical support workers	25 (4.84)	15 (5.59)	10 (4.02)		
Service and sales workers	69 (13.35)	35 (13.06)	34 (13.65)		
Skilled agricultural, forestry and fishery workers	64 (12.38)	34 (12.69)	30 (12.05)		
Craft and related trades workers, plant and machine operators and assemblers	40 (7.74)	19 (7.09)	21 (8.43)		
Other	124 (23.98)	57 (21.27)	67 (26.91)		
Missing	41 (7.93)	23 (8.58)	18 (7.23)		
Mother’s occupation					
Legislators, senior officials and managers	77 (14.89)	41 (15.30)	36 (14.46)	1.84	0.968
Professionals, technicians and associate professionals	53 (10.25)	30 (11.19)	23 (9.23)		
Clerical support workers	44 (8.51)	22 (8.21)	22 (8.84)		
Service and sales workers	77 (14.89)	39 (14.55)	38 (15.26)		
Skilled agricultural, forestry and fishery workers	62 (12.00)	30 (11.20)	32 (12.85)		
Craft and related trades workers, plant and machine operators and assemblers	15 (2.90)	9 (3.36)	6 (2.41)		
Other	148 (28.63)	74 (27.61)	74 (29.72)		
Missing	41 (7.93)	23 (8.58)	18 (7.23)		

Table 2 shows the estimated effects of maternal parenting on smartphone addiction before and after adjusting for potential individual and family covariates. In model 3, which adjusted for both individual and family characteristics, only maternal protection was found to be significantly associated with smartphone addiction (OR = 1.046; 95% CI:1.005-1.087). The significance of the regression relationship did not change before and after considering possible confounders (OR = 1.043; 95% CI:1.004-1.084 in model 1, and OR = 1.042; 95% CI:1.003-1.083 in model 2). Average marginal effects showed that, with a 1-point increase in maternal protection, medical students were 4.5 percentage points more likely to have smartphone addiction in model 3. In models 1 and 2, the probability of change in smartphone addiction were 4.2 percentage points and 4.1 percentage points respectively (see Table 2).

**Table 2:** Logistic regression analyses between maternal parenting and smartphone addiction (*n* = 517)

Variable	Smartphone addiction, OR (95% CI)		
	Model1^a^	Model2^b^	Model3^c^
Maternal care	0.974 (0.937,1.012)	0.972 (0.935,1.010)	0.976 (0.936,1.016)
Maternal protection	1.043 (1.004,1.084)*	1.042 (1.003,1.083)*	1.046 (1.005,1.087)*
Age		0.907 (0.717,1.148)	0.883 (0.690,1.129)
Gender-Male (ref)			
Female		0.965 (0.666,1.399)	0.979 (0.671,1.430)
Duration of medical program-5-year (ref)			
8-year		1.150 (0.776,1.706)	1.138 (0.720,1.799)
Household registration area-Rural (ref)			
Urban		0.978 (0.658,1.454)	1.170 (0.704,1.943)
Annual household income (¥)- ≤ 20,000 (ref)			
20,000-50,000			0.816 (0.467,1.427)
50,000-100,000			0.660 (0.390,1.118)
≥ 100,000			0.633 (0.258,1.555)
Mother’s educational level-Junior high school or below (ref)			
High school			0.902 (0.541,1.505)
University or above			0.855 (0.464,1.574)
Mother’s occupation-Legislators, senior officials and managers (ref)			
Professionals, technicians and associate professionals			0.931 (0.445,1.946)
Clerical support workers			1.026 (0.456,2.309)
Service and sales workers			1.058 (0.530,2.110)
Skilled agricultural, forestry and fishery workers			1.070 (0.471,2.429)
Craft and related trades workers, plant and machine operators and assemblers			0.636 (0.200,2.027)
Other			0.950 (0.487,1.853)
Log likelihood function	− 348.479	− 347.844	− 344.743
Pseudo R^2^	0.027	0.028	0.037
AIC	702.958	709.687	725.486
BIC	715.702	739.424	801.951
Marginal effect			
Maternal care	−0.027 (− 0.065,0.011)	−0.029 (− 0.068,0.010)	−0.025 (− 0.066,0.016)
Maternal protection	0.042 (0.004,0.080)*	0.041 (0.003,0.079)*	0.045 (0.005,0.084)*

*p < 0.05; **p < 0.01

^a^only maternal parenting variables were included

^b^maternal parenting variables and individual characteristics were included

^c^maternal parenting variables, individual characteristics, and family characteristics were included

Table 3 shows the logistic regressions between paternal parenting and smartphone addiction. In the paternal models, only paternal care was found to be significantly associated with lower odds of smartphone addiction after adjusting for all confounders (OR = 0.954; 95% CI:0.919-0.989 in model 3). The significance was consistent in all three models (OR = 0.952; 95% CI:0.918-0.986 in model 1, and OR = 0.951; 95% CI:0.917-0.985 in model 2). Average marginal effects showed that there was a 4.8-point decrease in the probability of experiencing smartphone addiction with a 1-point increase in level of paternal care. The probability of smartphone addiction did not change substantially with the gradual introduction of covariates (see Table 3).

**Table 3:** Logistic regression analyses between paternal parenting and smartphone addiction (*n* = 517)

Variable	Smartphone addiction, OR (95% CI)		
	Model1^a^	Model2^b^	Model3^c^
Paternal care	0.952 (0.918,0.986)**	0.951 (0.917,0.985)**	0.954 (0.919,0.989)*
Paternal protection	1.024 (0.986,1.063)	1.023 (0.985,1.063)	1.025 (0.986,1.065)
Age		0.902 (0.716,1.137)	0.884 (0.692,1.128)
Gender-Male (ref)			
Female		0.939 (0.648,1.363)	0.950 (0.648,1.393)
Duration of medical program-5-year (ref)			
8-year		1.157 (0.778,1.720)	1.152 (0.726,1.828)
Household registration area-Rural (ref)			
Urban		0.959 (0.646,1.423)	1.016 (0.635,1.626)
Annual household income(¥)- ≤ 20,000 (ref)			
20,000-50,000			0.860 (0.489,1.512)
50,000-100,000			0.733 (0.429,1.254)
≥ 100,000			0.696 (0.270,1.792)
Father’s educational level-Junior high school or below (ref)			
High school			0.782 (0.479,1.278)
University or above			1.007 (0.571,1.776)
Father’s occupation-Legislators, senior officials and managers (ref)			
Professionals, technicians and associate professionals			0.695 (0.357,1.354)
Clerical support workers			0.736 (0.289,1.877)
Service and sales workers			0.927 (0.459,1.872)
Skilled agricultural, forestry and fishery workers			0.844 (0.373,1.907)
Craft and related trades workers, plant and machine operators and assemblers			1.014 (0.431,2.388)
Other			1.044 (0.552,1.976)
Log likelihood function	−346.850	−346.116	−342.266
Pseudo R^2^	0.031	0.033	0.044
AIC	699.700	706.232	720.532
BIC	712.445	735.968	796.997
Marginal effect			
Paternal care	−0.050(−0.085,-0.014)**	−0.051(−0.087,-0.015)**	−0.048(−0.085,-0.011)*
Paternal protection	0.024(− 0.014, 0.061)	0.023(− 0.015,0.061)	0.025(− 0.014,0.063)

*p < 0.05; **p < 0.01

^a^only paternal parenting variables were included

^b^paternal parenting variables and individual characteristics were included

^c^paternal parenting variables, individual characteristics, and family characteristics were included

The interaction term of maternal care and protection was introduced into the logistic models to assess whether the associations between maternal protection and smartphone addiction were influenced by the level of maternal care (see Table 4). In model 3, the interaction term between maternal care and protection was statistically significant with smartphone addiction (OR = 1.005; 95% CI:1.001-1.009). Odds ratios remained almost the same before and after controlling for individual and family characteristics. These findings indicated that maternal care enhanced the estimated effects of maternal protection on smartphone addiction. That is, when the maternal care score increased, the estimated effect of maternal protection on smartphone addiction increased as well. Marginal effect analyses indicated that with the inclusion of the interaction between maternal care and protection, a 1-point increase in maternal protection score resulted in 4.4 percentage points increase in likelihood for smartphone addiction in medical students.

**Table 4:** Associations between maternal parenting variables, their interactions, and smartphone addiction (*n* = 517)

Variable	Smartphone addiction, OR (95% CI)		
	Model1^a^	Model2^b^	Model3^c^
Maternal care	0.917 (0.861,0.977)**	0.915 (0.859,0.975)**	0.919 (0.861,0.982)*
Maternal protection	0.924 (0.828,1.032)	0.922 (0.826,1.030)	0.927 (0.828,1.038)
Maternal care×protection	1.005 (1.001,1.009)*	1.005 (1.001,1.009)*	1.005 (1.001,1.009)*
Log likelihood function	− 345.879	−345.234	− 342.297
Pseudo R^2^	0.034	0.036	0.044
AIC	699.757	706.468	722.594
BIC	716.749	740.452	803.307
Marginal effect			
Maternal care	−0.033(−0.072,0.005)	−0.035(− 0.074,0.004)	− 0.031(− 0.072,0.009)
Maternal protection	0.042 (0.004,0.081)*	0.041 (0.003,0.080)*	0.044 (0.005,0.084)*

*p < 0.05; **p < 0.01

^a^only maternal parenting variables and their interaction term were included

^b^maternal parenting variables, their interaction term, and individual characteristics were included

^c^maternal parenting variables, their interaction term, individual characteristics, and family characteristics were included

Similarly, the interaction term of paternal care and protection was included to verify if paternal protection modified the relationship between paternal care and smartphone addiction (see Table 5). Regression results confirmed that the estimated effect of paternal care on smartphone addiction did not depend on paternal protection. As shown by marginal effect analysis after the inclusion of the interaction between paternal care and protection, a 1-point increase in paternal care score might lead to 4.7 percentage points decrease in the probability of smartphone addiction.

**Table 5:** Associations between paternal parenting variables, their interactions, and smartphone addiction (*n* = 517)

Variable	Smartphone addiction, OR(95% CI)		
	Model1^a^	Model2^b^	Model3^c^
Paternal care	0.917 (0.867,0.971)**	0.917 (0.867,0.970)**	0.918 (0.866,0.974)**
Paternal protection	0.936 (0.844,1.038)	0.937 (0.845,1.039)	0.935 (0.841,1.041)
Paternal care×protection	1.004 (0.999,1.008)	1.004 (0.999,1.008)	1.004 (0.999,1.008)
Log likelihood function	−344.976	−344.334	−340.467
Pseudo R^2^	0.036	0.038	0.049
AIC	697.952	704.669	718.935
BIC	714.944	738.653	799.648
Marginal effect			
Paternal care	−0.048(−0.086,-0.011)*	−0.049(−0.087,-0.011)*	−0.047(− 0.086,-0.008)*
Paternal protection	0.026(− 0.014,0.066)	0.025(− 0.014,0.065)	0.027(− 0.014,0.067)

*p < 0.05; **p < 0.01

^a^only paternal parenting variables and their interaction term were included

^b^paternal parenting variables, their interaction term, and individual characteristics were included

^c^paternal parenting variables, their interaction term, individual characteristics, and family characteristics were included

In the assessment of goodness of fit for both the maternal and paternal models, AIC was smaller after including the interaction term in the models, and BIC showed an inverse result. This indicated that the interaction terms did not change the fitness of the models.

## Discussion

To the best of our knowledge, this was the first study to explore the associations between parental bonding and smartphone addiction in Chinese medical students. Considering the different parental roles in child rearing among Chinese parents, we examined the estimated effects of parental bonding on smartphone addiction for mothers and fathers separately. Logistic regression models showed that maternal protection was positively associated with smartphone addiction, and paternal care was negatively associated with smartphone addiction. More detailed analyses showed that maternal care significantly enhanced the estimated effect of maternal protection on smartphone addiction.

Findings showed that 48.16% of the sample was categorized as having smartphone addiction, which, along with existing evidence of prevalent smartphone addiction, suggests a growing social issue [57]. The proportion was relatively higher than general university students in China (38.5%) [52]. This difference between medical students and other university students might be attributed to the higher stress levels that medical students face on a regular basis [58–60]. Compensatory Internet Use Theory depicts excessive internet use as a maladaptive strategy to cope with negative emotions [61], and these same pathways have also been demonstrated in smartphone addiction [62]. As third-year medical students in China begin their transition to specialized courses and clinical clerkship [63], they also take on a heavier academic load. Smartphone addiction, whether subconscious or intentional, might be a coping strategy to alleviate this academic pressure. Regarding the higher prevalence of smartphone addiction in medical students, interventions for different professions and undergraduate programs should be tailored, with much attention paid to those in the medical profession.

It is worth noting that there exist other studies that have outlined differences in characteristics between addictive and non-addictive groups [64–69]. However, related literature failed to provide consistent evidence of relationships between individual/family factors and addictive behaviors, which include both smartphone addiction and internet addiction. For example, in terms of gender, some report significant correlations between female gender and problematic usage [64, 70]. In contrast, some show being male increased the probability of smartphone addiction [71, 72], while others did not detect any gender difference [73, 74]. The sample of this study was mainly from the same grade, and the age variant was very limited. As a result, the correlation between age and smartphone usage was very weak. In addition, one primary objective of this research was to identify potential family effects on smartphone addiction. Our results confirmed that parental bonding—one essential family characteristic—was significantly associated with smartphone usage. Admittedly, the number of individual and family features investigated in one paper is very limited, and other important factors pertaining to the individual or to the family could be focused on in future research.

In this study, medical students with smartphone addiction tended to relate differently with their mothers and fathers. Namely, students with overprotective mothers or indifferent fathers tend to score higher on the smartphone addiction questionnaire. Similarly, differences in estimated effects of paternal and maternal parenting on problematic internet use [75] and psychosocial functioning were also found in other previous studies involving mood disorders [43] and antisocial personality traits [76].

The difference may be due to the different roles and responsibilities of mothers and fathers in Chinese families. In the Chinese culture, mothers usually play the dominant role in child rearing, showing greater investment than fathers [77]. And children often report high levels of both positive and negative maternal parenting attributes, including both adequate care and responsiveness as well as harshness and control [77–79]. Therefore, it is not unusual that maternal day-to-day parenting also has a greater impact on shaping children’s behaviors [79]. On the other hand, despite the predominant role of mothers, fathers are becoming more and more involved in parenting [80] and have been regarded as the head of the family. As such, paternal care, in the form of emotional warmth, had been found to be more important in adolescents’ social development [78].

While there is a plethora of publications on parenting and addiction, few have specifically examined maternal parenting and its effects. In this study, maternal protection was found to be significantly associated with smartphone addiction. This was similar to a former study conducted among Chinese college students, which demonstrated that parental overprotection and rejection were correlated with smartphone addiction [24]. Students who have a history of maternal overprotection may be subject to problems [24, 81] such as lower morality, poor interpersonal relationships, lack of self-control, low self-efficacy, and internalizing and externalizing problems, all of which are influencing factors to smartphone addiction. Additionally, children with overprotective mothers might also experience lower levels of early life stressors. These children may be more sensitive to encountered stressors compared to those who have been exposed to higher levels of early life stressors [82], and there is positive evidence of smartphone addiction being linked with stress [83]. Moreover, being addicted with smartphones could be seen as a way for children to break free from parental overprotection and to seek autonomy [39], and the previous discussion on related addictions, such as internet addiction, had been considered to be less harmful and more available than other illegal substances [84].

Contrary to a previous study that demonstrated maternal overprotection combined with inadequate care to be a risk factor of problematic internet use in adolescents [85], our study found that maternal care significantly enhanced the estimated effects of maternal protection on smartphone addiction. This may be caused by maladjustment to university life and worse social functioning due to students’ dysfunctional attitudes toward dependency. Maternal overprotection might hinder the psychological adjustment of young adult children [86], and the combination of maternal overprotection and care might aggravate children’s dependency on mothers, as seen in the hypotheses of Bowlby [31] which demonstrated that insecure attachment in infants was associated with higher dependence on others in later life. As previously mentioned by Bowlby [30–32] and Ainsworth et al. [33], children internalize parental bonding styles into working models, which mostly remain constant throughout the lifetime and persistently influence their cognition, emotions, and behaviors. The combination of maternal care and overprotection (affectionate constraint) was found to be associated with impaired formation of positive working models [87] that characterize self and others as effective and worthy. Individuals with insecure working models may be susceptible to mental disorders in stressful conditions [87], and such high-stress environments are common in medical education.

Our study found that paternal care was negatively associated with smartphone addiction. This was in line with previous studies which found that lower levels of paternal care was linked with problematic internet use [75] and smartphone addiction [88], Paternal care could play an indirect role on smartphone addiction through impact on family cohesion and enmeshed family functioning [88]. A study conducted in China revealed that inadequate paternal care has a significant negative association with adolescents’ social development, and the association was stronger than that of maternal care [78]. Students with inadequate social engagement and social networks perceived less comfortable social interactions and real-life support and were more likely to be addicted to their smartphones [25]. Moreover, low paternal care was also considered as a determinant of mental disorders [78, 89]. Students with mental disorders may exhibit signs of smartphone addiction as a coping mechanism [62, 64].

In examining the differences in perception of parental bonding across cultures, it would be best not to make direct comparisons of the raw PBI score. It is worth noting that cross-cultural differences between Western and Eastern countries may affect the perceptions of parental bonding. The way of conveying affection to children differs greatly between Western countries and East Asian countries. Western parents often show outward affection, such as kissing and praising, while Asian parents show their warmth via devotion and close monitoring [90]. The Western culture mostly advocates individuality and freedom, while Eastern cultures emphasize collectivism [91]. Liu et al. have indicated that children of Eastern cultures may take a neutral stance on autonomy [92], and thus attitudes toward parental overprotection and its potentially adverse impacts in Eastern populations may not appear as negative as those from Western countries. Moreover, both parental discipline and governing are synonymous with involvement and care in China [93]. As was demonstrated in Korea [94], there is also a high possibility that children perceive maternal protection as care in the Chinese culture.

There were several limitations in this study. Firstly, due to the retrospective self-reported nature of this survey by students who have already reached adulthood, there may be some degrees of recall bias. Secondly, a single institution was surveyed in this study, which might not be representative of all medical students in China. Thirdly, the underlying mechanisms of parental bonding and smartphone addiction were not well explored in this study. Future studies should further investigate the psychological and social pathways of this association and would benefit from investigating a nationally representative sample and exploring the differences across grade levels.

## Conclusion

In summary, this study examined the relationships between maternal/paternal parenting and smartphone addiction in Chinese medical students and revealed the high prevalence of smartphone addiction among Chinese medical students. Importantly, associations between parental bonding and smartphone addiction in Chinese medical students differed for mothers and fathers. Maternal protection increased the probability of smartphone addiction, and maternal care enhanced the estimated effect of protection on smartphone addiction. On the contrary, paternal care was associated with lower odds of smartphone addiction. Medical educators seeking to implement smartphone addiction interventions or to mitigate the potential negative effects of smartphone addiction in their medical students may want to pay closer attention to students who have experienced maternal overprotection and poor paternal care.

## Data Availability

The datasets used and analysed during the current study are available from the corresponding author upon reasonable request.
